# Ablation of the canonical testosterone production pathway via knockout of the steroidogenic enzyme HSD17B3, reveals a novel mechanism of testicular testosterone production

**DOI:** 10.1096/fj.202000361R

**Published:** 2020-06-18

**Authors:** Diane Rebourcet, Rosa Mackay, Annalucia Darbey, Michael K. Curley, Anne Jørgensen, Hanne Frederiksen, Rod T. Mitchell, Peter J. O’Shaughnessy, Serge Nef, Lee B. Smith

**Affiliations:** ^1^ School of Environmental and Life Sciences University of Newcastle Callaghan NSW Australia; ^2^ MRC Centre for Reproductive Health University of Edinburgh The Queen’s Medical Research Institute Edinburgh UK; ^3^ Department of Growth and Reproduction, Rigshospitalet University of Copenhagen Copenhagen Denmark; ^4^ International Centre for Research and Research Training in Endocrine Disruption of Male Reproduction and Child Health, Rigshospitalet University of Copenhagen Copenhagen Denmark; ^5^ Institute of Biodiversity, Animal Health, and Comparative Medicine University of Glasgow Glasgow UK; ^6^ Department of Genetic Medicine and Development Faculty of Medicine University of Geneva Geneva Switzerland

**Keywords:** androgens, HSD17B3, HSD17B12, Leydig cell, testis, testosterone

## Abstract

Male development, fertility, and lifelong health are all androgen‐dependent. Approximately 95% of circulating testosterone is synthesized by the testis and the final step in this canonical pathway is controlled by the activity of the hydroxysteroid‐dehydrogenase‐17‐beta‐3 (HSD17B3). To determine the role of HSD17B3 in testosterone production and androgenization during male development and function we have characterized a mouse model lacking HSD17B3. The data reveal that developmental masculinization and fertility are normal in mutant males. Ablation of HSD17B3 inhibits hyperstimulation of testosterone production by hCG, although basal testosterone levels are maintained despite the absence of HSD17B3. Reintroduction of HSD17B3 via gene‐delivery to Sertoli cells in adulthood partially rescues the adult phenotype, showing that, as in development, different cell‐types in the testis are able to work together to produce testosterone. Together, these data show that HS17B3 acts as a rate‐limiting‐step for the maximum level of testosterone production by the testis but does not control basal testosterone production. Measurement of other enzymes able to convert androstenedione to testosterone identifies HSD17B12 as a candidate enzyme capable of driving basal testosterone production in the testis. Together, these findings expand our understanding of testosterone production in males.

**Abbreviations**
17‐OHP: 17‐OH‐progesterone
AGD: anogenital distance
Amh: anti‐mullerian hormone
AKR: aldo‐keto reductase
AR: androgen receptor
*Cyp11a1*: cytochrome P450 11A1, cholesterol side‐chain cleavage enzyme
*Cyp17a1*: cytochrome P450 17A1
D4: androstenedione
DDX4: DEAD‐box helicase 4
Dhh: desert hedgehog
GFP: green fluorescent protein
GnRH: gonadotrophin releasing hormone
hCG: human chorionic gonadotrophin
hpg: hypothalamus‐pituitary‐gonadal axis
HSD17B: hydroxysteroid‐dehydrogenase‐17‐beta
HSD3B1: hydroxysteroid‐dehydrogenase‐3‐beta
LH: luteinizing hormone
*Lhcgr*: luteinizing hormone/choriogonadotropin receptor
Lv GFP: GFP control lentiviral particles
Lv *Hsd17b3*: *Hsd17b3* lentiviral particles
SDR: short‐chain dehydrogenase/reductase enzymes
SOX9: SRY‐box 9
*StAR*: steroidogenic acute regulatory protein
T: testosterone

## 1 INTRODUCTION

Male development, fertility, and lifelong health and wellbeing are all androgen‐dependent. Perturbed androgen action at any stage of life, but particularly during aging,^1^ significantly impacts the quality of life, and low androgens are an independent risk factor for all‐cause early death.^2^ The long‐established paradigm of androgen action identifies testosterone (T) as the key androgen driving male development and function. The final step in this canonical pathway is controlled by the essential activity of a single testis‐specific 17‐ketosteroid reductase enzyme, hydroxysteroid dehydrogenase 17‐beta 3 (HSD17B3). During development in mice, HSD17B3 is expressed in testicular Sertoli cells where it functions to produce testosterone from androstenedione derived from the fetal Leydig cells. Expression of HSD17B3 switches from Sertoli cells in fetal life to the adult Leydig cell population in prepubertal life that produces testosterone directly.^3^, ^4^

The importance of HSD17B3 for testicular testosterone production in humans is highlighted in differences/disorders of sex development involving mutations in *HSD17B3* that reduce its function. Individuals with perturbed HSD17B3 function are under‐masculinized at birth, with hypoplastic to normal internal genitalia (epididymis, vas deferens, seminal vesicles, and ejaculatory ducts), but with female external genitalia and the absence of a prostate.^5^, ^6^, ^7^ Inadequate testosterone production by the testis reduces the availability of dihydrotestosterone in peripheral tissues leading to under‐masculinization of the external genitalia during development. During puberty, increased luteinizing hormone (LH) stimulation of testicular androgen production leads to an increase in production and secretion of androstenedione (the precursor of testosterone). A diagnostic marker of HSD17B3 deficiency is, therefore, a high androstenedione to testosterone ratio in the blood.^8^ Conversion of androstenedione to testosterone/dihydrotestosterone in peripheral tissues promotes masculinization at puberty, failure of which is another diagnostic marker of HSD17B3 deficiency.

The presence or absence of male internal reproductive organs and external genitalia is critically dependent upon signaling via the androgen receptor (AR) during the masculinization programming window in fetal life.^9^ Stimulation of AR with androgens prior to this window does neither induce premature masculinization,^10^ nor can stimulation with androgens after this window has closed, drive the establishment of male internal genitalia if AR is blocked during the window.^9^ During the programming window, testicular testosterone is the androgen that drives internal masculinization and it is curious, therefore, that some individuals lacking HSD17B3 show signs of internal masculinization during fetal life.^11^ This may reflect the nature of the specific mutations that reduce, but do not completely ablate, testosterone production in the testis. Alternatively, it may suggest the presence of another mechanism driving testosterone production that does not require the function of HSD17B3.

To address this, we have characterized a mouse model in which HSD17B3 has been completely ablated (complete knockout model). Consistent with the range of diagnoses in humans lacking HSD17B3, knockout mice develop normal internal genitalia at birth. In adulthood, as with humans lacking HSD17B3, knockout mice also display a high androstenedione to testosterone ratio. Surprisingly, however, HSD17B3 knockout mice continue to produce testosterone, display normal spermatogenesis and are fertile, something that is widely accepted to be fundamentally dependent upon the 17‐ketosteroid reductase activity of HSD17B3 and its ability to produce testosterone. Together, these data identify an as yet uncharacterized, alternative mechanism driving testosterone production in the testis that functions independently of HSD17B3, which has implications for our understanding of testosterone production in males.

## 2 MATERIALS AND METHODS

### 2.1 Breeding of transgenic mice

The knockout model *Hsd17b3* ^tm(lacZ;neo)lex^ was provided by Lexicon, Taconic, and generated using classical gene targeting approaches. A selection cassette was inserted into exon 1 (chromosome 13) to generate a subsequent frameshift mutation and was undertaken on genetic background 129/svEv‐C57bl/6. Heterozygous *Hsd17b3* ^tm(lacZ;neo)lex^; +/− (referred as *Hsd17b3* ^+/−^) males and females were bred together to generate *Hsd17b3* ^+/+^, *Hsd17b3* ^+/−^, and *Hsd17b3* ^−/−^ animals. Animals were identified by genotyping from ear or tail DNA for the presence of *Hsd17b3* and the presence of mutated allele (Neo) using standard PCR with appropriate primers (Table 1). Mice were housed under standard conditions of care and bred as described in the results. Experiments passed local ethical review and were conducted with licensed permission under the UK Animal Scientific Procedures Act (1986), Home Office license number PPL 70/8804 and in accordance with the University of Newcastle's Animal Care Ethics Committee guidelines (Approval No. A‐2018‐820).

**TABLE 1:** Primer list

Gene	Forward primer	Reverse primer	Probe
*Hsd17b3 (WT)*	gatgcctgcgaatcacaag	ccattgatcgcaggaaagag	genomic
*Hsd17b3 (mutant neomycin)*	gcagcgcatcgccttctatc	gtgaatagaggcacagaatgc	genomic
*Lhcgr*	gatgcacagtggcaccttc	cctgcaatttggtggaagag	UPL #107
*Star*	aaactcacttggctgctcagta	tgcgataggacctggttgat	UPL #83
*Cyp11a1*	aagtatggccccatttacagg	tggggtccacgatgtaaact	UPL #104
*Hsd3b1*	gaactgcaggaggtcagagc	gcactgggcatccagaat	UPL #12
*Cyp17a1*	catcccacacaaggctaaca	cagtgcccagagattgatga	UPL #67
*Hsd17b3*	atgggcagtgattaccggagca	tacaatcttcacacagcttccagtggtc	UPL #47
*Hsd17b1*	ccccacggtagtgctcatt	ccgcaatgtggcataaact	UPL #82
*Hsd17b5*	ccattggggtgtccaactt	ccagaagttttccctgattga	UPL #27
*Hsd17b12*	cgtggaatgaagattgtcctg	cagcaatggtccttgtttca	UPL #62
*Luciferase*	gcacatatcgaggtgaacatcac	gccaaccgaacggacattt	5’NED‐tacgcggaatacttc

### 2.2 Trophic stimulation

To examine the testicular response to acute trophic stimulation in vivo, adult animals were given a single IP injection of 20IU human chorionic gonadotrophin hCG (Pregnyl, Organon), and then, sacrificed 16 hours later. Serum was collected for measurement of testosterone levels.

### 2.3 Lentiviral production

Lentiviral particles contained CMV‐mouse *Hsd17b3* and GFP (emGFP) transgenes separated with an IRES site or CMV‐emGFP alone. Shuttle vectors were packaged with a third generation Lentiviral vector plasmid pseudotyped for VSV‐G, concentrated to a viral titer of > 5 × 10^9^ TU/mL in serum free media.^12^

### 2.4 Lentiviral injection

Procedures were performed under anesthesia by inhalation of isoflurane and under aseptic conditions. In brief, the lower abdomen was shaved and sterilized before a small incision was made in the abdominal muscle wall. Testes were exposed through the incision and, under a dissection microscope, the efferent duct and areas around the rete testis were isolated from surrounding adipose tissue taking care not to rupture blood vessels and to keep the testes moist at all times with sterile saline. Up to 10 µL of the lentiviral particle suspension were introduced into the seminiferous tubules of adult (day 120) *Hsd17b3* ^−/−^ males and control littermates via the rete testis (similar to techniques reported by Ref. [13]) using a glass micropipette (outer diameter: 80 µm, beveled) (Biomedical Instruments, Germany) and a microinjector (Eppendorf Femtojet; Eppendorf, Germany) at a pressure of 25 hPA. Delivery of particles was monitored by the addition of Trypan Blue dye to the viral particles (0.04%). Testes were then carefully replaced back into the abdominal cavity, taking care to ensure access to the scrotal compartment was present, and incisions were closed using sterile sutures. Mice were injected subcutaneously with 0.05 mg/kg buprenorphine (Vetergesic; Ceva Animal Health Ltd, UK) while anesthetized and allowed to recover on a heat pad while being monitored. Animals were culled (as described below) and testis recovered 7 weeks postsurgery and imaged in cold phosphate‐buffered saline with a Leica MZFLIII microscope (Wetzlar, Germany) and an epifluorescent green fluorescent protein (GFP) filter. Testes were weighed and fixed separately in Bouins fixative (Clin‐Tech Ltd, UK) for 6 hours before being transferred to 70% of ethanol prior to processing into paraffin wax and sectioned at 5 µm for histological analysis.

### 2.5 Tissue collection

Animals were culled at different key points of testis development by CO_2_ inhalation and subsequent cervical dislocation. Blood was obtained by cardiac puncture for hormonal profile analysis. Plasma was separated by centrifugation and stored at −80°C. Bodyweight and reproductive organ (testis and seminal vesicle) weights were recorded. Collected tissues were either fixed or frozen for RNA or protein analysis.

### 2.6 Quantitative RT‐PCR

Quantitative RT‐PCR was performed as previously described^14^ with minor modifications. RNA concentration was estimated using a NanoDrop One spectrophotometer (Thermo Fisher Scientific) and cDNA was prepared using the SuperScript VILO cDNA synthesis Kit (Invitrogen). cDNA quality was assessed using Universal ProbeLibrary Mouse ACTB Gene Assay (Sigma). Real‐time PCR was carried out on the ABI Prism 7900HT Real‐Time PCR System (Applied Biosystems) in 384‐well format using TaqMan Universal PCR Master Mix (Applied Biosystems) and the Universal Probe Library (Roche).^14^ Details of each assay are listed in Table 1.

### 2.7 Immunostaining

Tissues were fixed in Bouins for 6 hours, stored in 70% of ethanol, and embedded in paraffin or in resin (see below). Sections were processed as previously described.^15^ Succinctly slides were dewaxed and rehydrated prior antigen retrieval in 0.01 M citrate buffer (pH 6.0). Successive steps to reduce the endogenous peroxidases activity and to block the nonspecific activity were undertaken and followed by incubation overnight at 4°C with the primary antibodies Table 2. After washing, slides were incubated for 30 minutes at room temperature with the appropriate secondary antibody conjugated to peroxidase. Sections were incubated with fluorescein Tyramide Signal Amplification system (“TSA”, Perkin Elmer) according to manufacturer's instructions. Sections were counterstained in Sytox Green (Molecular Probes, life technologies, Paisley, UK) and mounted in PermaFluor mounting medium (Thermo Scientific, UK). Slides were imaged using a LSM 710 confocal microscope and ZEN 2009 software (Carl Zeiss Ltd, Hertfordshire, UK). Control sections were incubated with no primary antibody. At least three different animals from each group were tested and processed simultaneously.

**TABLE 2:** Details of antibodies and detection methods used

Primary antibody (AbI) name	References	Dilution AbI	RRID	Secondary antibody (AbII) conjugated		Dilution AbII	Detection system
HSD17B3	Santa Cruz sc‐135044	1/2000	AB_10658251	Peroxidase	Vector Laboratories PI‐1000	1/200	IF
SOX9	Millipore Ab5535	1/8000	AB_2239761	Peroxidase	Vector Laboratories PI‐1000	1/200	IF
3 β‐HSD	Santa Cruz Biotechnology sc‐30820	1/750	AB_2279878	Peroxidase	Santa Cruz Biotechnology sc‐2961	1/200	IF
CYP17A1	Santa Cruz Biotechnology sc‐46081	1/1000	AB_2088659	Peroxidase	Santa Cruz Biotechnology sc‐2961	1/200	IF
DDX4	Abcam Ltd. Ab13840	1/400	AB_443012	Peroxidase	Vector Laboratories PI‐1000	1/200	IF
CL.CASPASE 3	Cell signalling (NEB) #9661	1/500	AB_2341188	Peroxidase	Vector Laboratories PI‐1000	1/200	IF
Green Fluorescent Protein (enhanced) (eGFP)	Abcam ab6556	1/200	AB_305564	Peroxidase	Vector Laboratories PI‐1000	1/200	IF
HSD17B12	LSBio LS‐B16612	1/100	Not classified	ImmPRESS HRP Goat Anti‐Rabbit IgG Polymer Detection Ki	Vector Laboratories MP‐7451		IHC

Abbreviations: IF, immunofluorescence; IHC, immunohistochemistry.

### 2.8 Stereology and histology

For stereology, testes were embedded in Technovit 7100 resin, cut into 20 µm sections, and stained with Harris’ hematoxylin, as previously described in Ref. [15]. The number of Leydig cells and Sertoli cells was determined by the optical disector method using an Olympus BX50 microscope fitted with a motorized stage (Prior Scientific Instruments, Cambridge, UK) and Stereologer software (Systems Planning Analysis, Alexandria, VA).

### 2.9 Intratesticular steroid extraction

A fragment of frozen testis was weight and homogenized in lysis buffer (50 mM Tris pH 7.4, 1% of deoxycholate, 0.1% of SDS). Steroids were extracted from testis lysate or from plasma using diethyl ether, dried under a constant stream of nitrogen, and then, resuspended in the appropriate buffer for analysis.

### 2.10 Hormone analysis

Concentrations of the gonadotropins luteinizing hormone (LH) and follicle‐stimulating hormone (FSH) were assessed using the Milliplex Map Pituitary Magnetic Bead Panel Kit (PPTMAG‐86K; MilliporeSigma, Burlington, MA, USA) inter‐assay cv <20%, intra‐assay cv <15%, range 24.4‐100 000 pg/mL for FSH and 4.9‐20 000 pg/mL for LH according to the manufacturer's instructions. A Bio‐Plex 200 suspension array system was used to measure plasma concentrations and data were analyzed with Bio‐Plex Manager software (Bio‐Rad Laboratories, Hercules, CA, USA). Our experimental intra CV for FSH assay <6.5 and for LH assay <3.4.

The steroids in mouse plasma and testis lysate were measured using an isotope‐dilution TurboFlow liquid chromatography‐tandem mass spectrometry method as previously described.^14^, ^16^ For progesterone, 17‐OH‐progesterone (17‐OHP), androstenedione, and testosterone the limits of quantification were 0.036 nM, 0.1 pM, 0.012 nM, and 0.042 nM, respectively, and the interday variation, expressed as the relative standard deviation for these analytes were testosterone was ≤5.3% and ≤4.2% for low and high spike levels of control materials, respectively, included three times each in every analytical batch. Chemical analyses were performed at the Dept. of Growth and Reproduction, Rigshospitalet, Copenhagen University Hospital.

### 2.11 Image analysis

Histological slides were analyzed and photographed using a Provis microscope (Olympus Optical, London, UK) fitted with a DCS330 digital camera (Eastman Kodak, Rochester, NY). For immunofluorescence, slides were imaged using a LSM 710 confocal microscope and ZEN 2009 software (Carl Zeiss Ltd, Hertfordshire, UK). Images were compiled using Adobe Photoshop CS6 and Adobe Illustrator 2019 (Adobe System Inc, Mountain View, CA, USA).

### 2.12 Statistical analysis

Data were analyzed using Graph Prism version 8 (GraphPad Software Inc, San Diego, CA, USA). Statistical analyses involved Student *t* test or one‐ or two‐way ANOVA with the appropriate post hoc tests (Tukey's multiple comparisons or Dunnett's tests). When required, data were normalized using log transformation. Values are expressed as means ± SEM *P < .05, **P < .01, ***P < .001, ****P < .0001.

## 3 RESULTS

### 3.1 HSD17B3 is localized to Sertoli cells in fetal life and Leydig cells in adulthood

**FIGURE 1:**
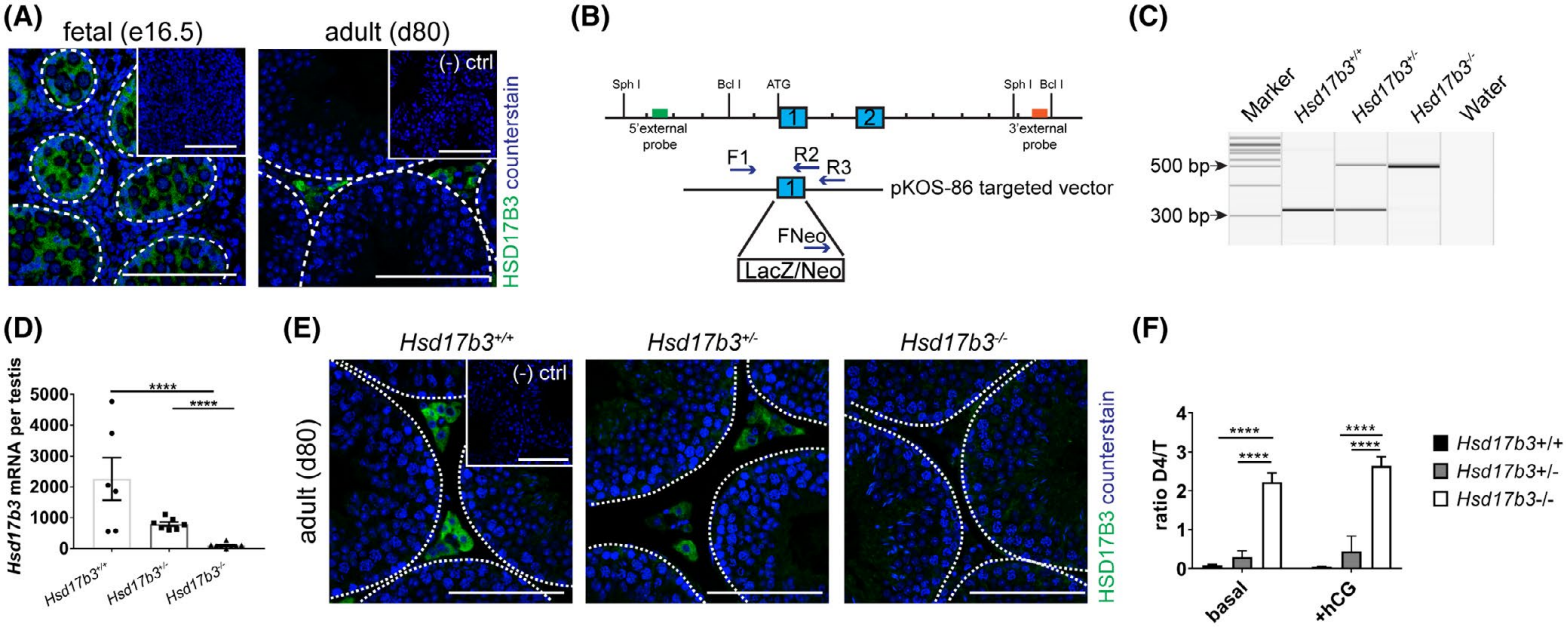
Validation of the HSD17B3 loss of function model. A, The spatiotemporal expression of HSD17B3 in wild‐type fetal mouse testis (e16.5) shows expression is restricted to Sertoli cells at e16.5 (inset shows absence of staining in e16.5 fetal testis using a mouse model depleted of Sertoli cells). In the wild‐type adult testis, HSD17B3 localizes to the Leydig cells (insert shows negative control) (bar: 100 µm). B, Schematic model of the knock‐in in *Hsd17b3* exon 1, detailing the localization of the primers on WT and mutant alleles. C, PCR analysis of the genomic DNA isolated from ear clips for *Hsd17b3* ^+/+^ (317 bp), *Hsd17b3* ^+/−^ (317 bp and 522 bp), and *Hsd17b3* ^−/−^ (522 bp) alleles. D, Relative expression of *Hsd17b3* transcripts, normalized to exogenous luciferase, shows a significant reduction in the expression of *Hsd17b3 in Hsd17b3* ^−/−^ testes (n = 6 *Hsd17b3* ^+/+^, n = 7 *Hsd17b3* ^+/−^, n = 8 *Hsd17b3* ^−/−^, ANOVA, ****P < .0001). E, Immunolocalization of HSD17B3 in adult testis shows an absence of staining in *Hsd17b3* ^−/−^ compared to *Hsd17b3* ^+/+^ and *Hsd17b3* ^+/−^ **mice (scale bar: 100 µm). F, Ratio of circulating levels of androstenedione (D4) to testosterone (T) at d80 under both basal and hyper‐stimulated (+hCG) conditions (n = 11(basal) n = 3 (+hCG) *Hsd17b3* ^+/+^, n = 13 (basal) n = 8 (+hCG) *Hsd17b3* ^+/−^, n = 12 (basal) n = 6 (+hCG) *Hsd17b3* ^−/−^, ANOVA ****P < .0001)

Expression of HSD17B3 has been reported to occur in different cell‐types depending on the stage of development of the testis.^3^, ^17^ Using immunofluorescence to localize HSD17B3 in fetal and adult testes, we confirmed that expression of this enzyme is restricted to Sertoli cells at e16.5, (Figure 1A) (and this was further verified using a mouse model that lacks Sertoli cells, Figure 1A insert)^15^ while, in adulthood, the expression is restricted to the Leydig cells (Figure 1A). Taken together the data confirm the previously published data describing the spatiotemporal expression profile of HSD17B3.^3^, ^17^

### 3.2 Disruption of exon 1 of *Hsd17b3* produces a HSD17B3 loss of function model

To understand the importance of HSD17B3 function in male development and fertility, we characterized a transgenic mouse model, in which exon 1 of the *Hsd17b3* gene had been disrupted via insertion of a LacZ/Neo expression cassette (Figure 1B,C). Interrogation of testicular cDNA by q‐RTPCR confirmed the absence of *Hsd17b3* transcripts in *Hsd17b3* ^−/−^ mice (Figure 1D) and lack of detectable HSD17B3 protein in the testis (Figure 1E). As with cases of HSD17B3 deficiency in humans, there was a marked increase in the androstenedione to testosterone (D4/T) ratio in *Hsd17b3* ^−/−^ mice under both basal and hyper‐stimulation (+hCG) conditions (Figure 1F). Together these data confirm the successful production of a mouse model lacking HSD17B3.

### 3.3 Blocking of the canonical testosterone production pathway via ablation of HSD17B3 function does not impact the development of the internal male genitalia

**FIGURE 2:**
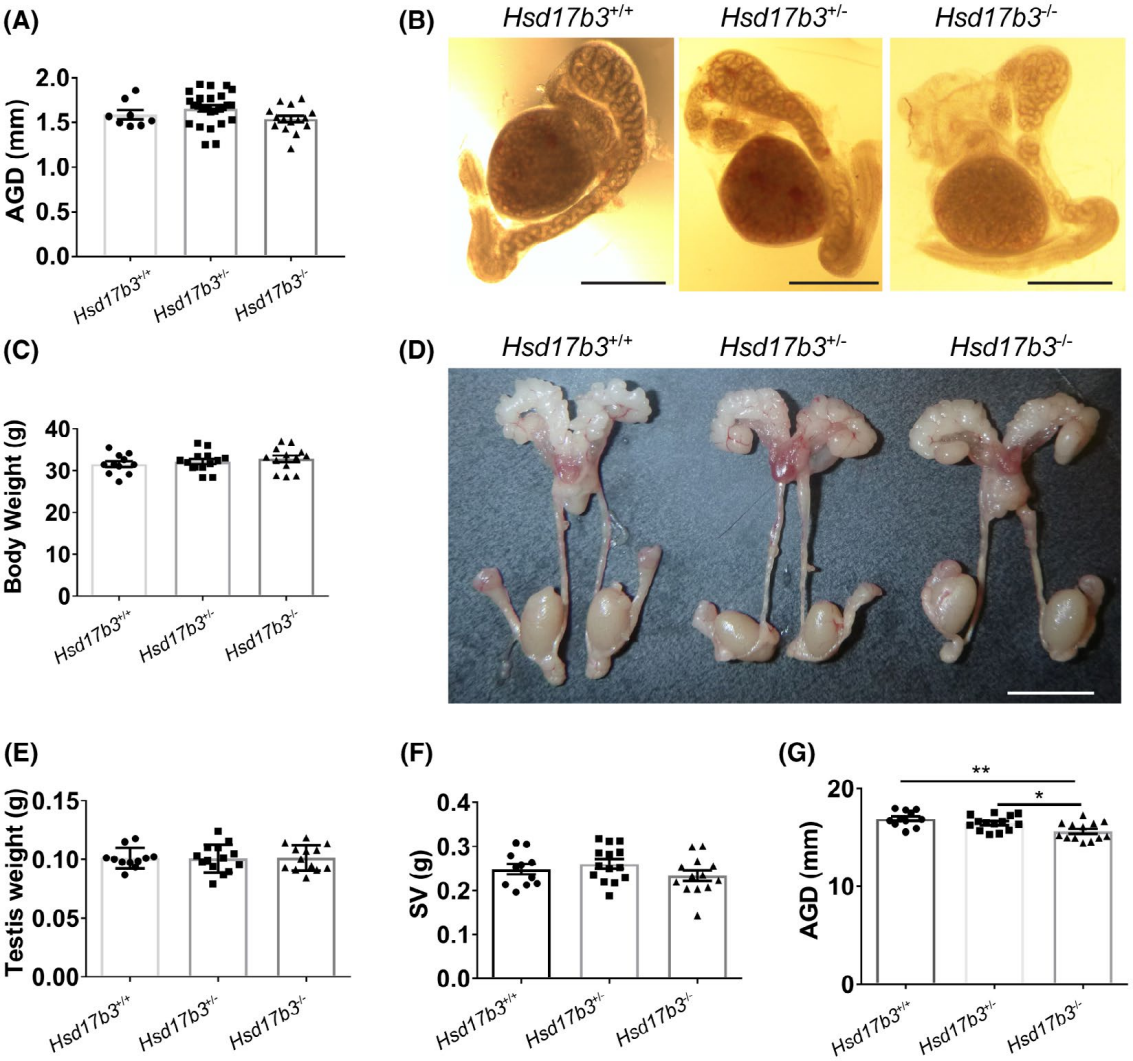
HSD17B3 loss of function does not impact the development of internal male genitalia. A, The anogenital distance in neonatal (d0) males did not change following HSD17B3 loss of function (n = 8 *Hsd17b3* ^+/+^, n = 26 *Hsd17b3* ^+/−^, n = 15 *Hsd17b3* ^−/−^, ANOVA). B, The gross morphology of the genitalia in neonatal (d0) *Hsd17b3* ^−/−^ animals showed normal epididymal coiling. C, Bodyweight in adult (d80) did not differ between genotypes (n = 11 *Hsd17b3* ^+/+^, n = 14 *Hsd17b3* ^+/−^, n = 13 *Hsd17b3* ^−/−^, ANOVA). D, The gross morphology of the reproductive system in adult (d80) *Hsd17b3* ^−/−^ animals was comparable to controls littermates. Testis (E) and seminal vesicles (F) weights were unchanged in *Hsd17b3* ^−/−^ animals at d80 (n = 11 *Hsd17b3* ^+/+^, n = 14 *Hsd17b3* ^+/−^, n = 13 *Hsd17b3* ^−/−^, ANOVA). G, Anogenital distance in adult (d80) males was significantly reduced following HSD17B3 loss of function (n = 11 *Hsd17b3* ^+/+^, n = 14 *Hsd17b3* ^+/−^, n = 13 *Hsd17b3* ^−/−^, ANOVA *P < .05, **P < .01)

To assess the impact on male development following the ablation of HSD17B3 function, we first analyzed the gross morphology of the genitalia and wider reproductive system in both neonatal (postnatal day 0: d0) and adult (d80) *Hsd17b3* ^−/−^ animals. At birth, anogenital distance (AGD, a biomarker of androgen action during the masculinization programming window^9^ was normal in *Hsd17b3* ^−/−^ males (Figure 2A). Consistent with this, other androgen‐dependent endpoints also developed normally: the initial segment of the epididymis was present^18^ and Wolffian duct/epididymis coiled normally^19^ in *Hsd17b3* ^+/−^ and *Hsd17b3* ^−/−^ animals (Figure 2B). In adulthood, there was no difference in body weight between the genotypes at d80 (Figure 2C) and the gross reproductive system of *Hsd17b3* ^−/−^ males appeared unchanged with respect to control animals (Figure 2D). Importantly, two key biomarkers of androgen action, testis weight (a biomarker of internal testicular androgens) (Figure 2E) and seminal vesicle weight (a biomarker of circulating androgens) (Figure 2 F) did not differ from wild‐type or heterozygous controls, suggesting that androgen signaling is not impacted at d80. However, a small reduction in AGD was observed in d80 *Hsd17b3* ^−/−^ males (Figure 2G), suggestive of a mild perturbation to androgen signaling in adulthood.^20^ As no difference was observed in any endpoint between wild‐type and heterozygous these groups were combined for downstream analyses and termed ‘controls’.

### 3.4 Blocking of the canonical testosterone production pathway does not alter male fertility

**FIGURE 3:**
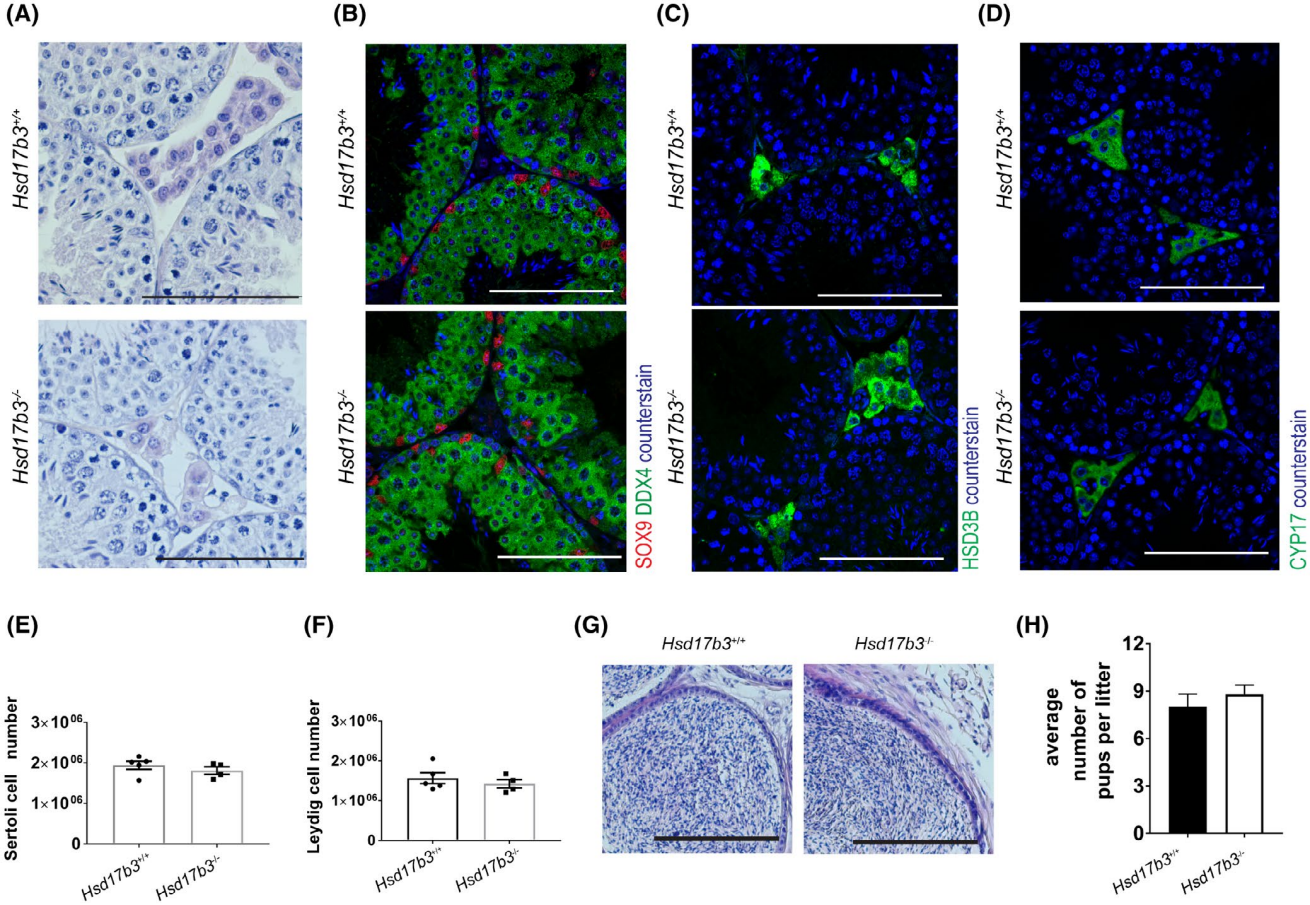
HSD17B3 loss of function does not alter male fertility. A, Testicular histology was normal in *Hsd17b3* ^−/−^ compared to *Hsd17b3* ^+/+^ animals (bar: 100 µm). Cell composition of the testis using immunofluorescence staining specific for (B) Sertoli cells and germ cells (SOX9 and DDX4, respectively) and (C) Leydig cells (HSD3B and CYP17A1) were normal in *Hsd17b3* ^−/−^ males (bar in B‐D: 100 µm). E, Sertoli and (F) Leydig cell numbers were unchanged in *Hsd17b3* ^−/−^ compared to *Hsd17b3* ^+/+^ animals (n = 5 *Hsd17b3* ^+/+^ and n = 4 *Hsd17b3* ^−/−^, *t*‐test). G, The cauda epididymis in *Hsd17b3* ^−/−^ animals contains spermatozoa (bar: 100 µm) and (H) *Hsd17b3* ^−/−^ males are fertile and produce normal numbers of pups per litter (n = 4‐5 per genotype, *t*‐test)

To determine the impact of the loss of HSD17B3 function on the adult testis, we first assessed the histology and cell composition of the testis. We undertook hematoxylin‐eosin staining and immunofluorescence localization using specific Sertoli cell (SOX9), germ cells (DDX4), and Leydig cell (HSD3B and CYP17A1) markers at d0 and in adulthood. At both time‐points the testicular histology, cell localization, and composition of different cell types (including all stages of germ cell development) were normal in *Hsd17b3* ^−/−^ animals (Figure 3A‐D and supplemental Figure S1). Similarly, numbers of Sertoli cells and Leydig cells in adulthood did not differ between control and *Hsd17b3* ^−/−^ males (Figure 3E‐F). As the overall composition of the testis in *Hsd17b3* ^−/−^ males was normal, we next assessed whether these males, lacking HSD17B3 function, were fertile. Upon analysis, the cauda epididymes in *Hsd17b3* ^−/−^ animals were found to contain abundant spermatozoa (Figure 3G) and when *Hsd17b3* ^−/−^ males were bred, viable pregnancies resulted, with no significant difference in the number of pups born compared to matings using wild‐type littermate controls as studs (Figure 3H). These data show that overall testicular development and fertility are not altered following the loss of HSD17B3.

### 3.5 Blocking of the canonical testosterone production pathway promotes a functional response within Leydig cells to maintain normal basal testosterone concentrations

**FIGURE 4:**
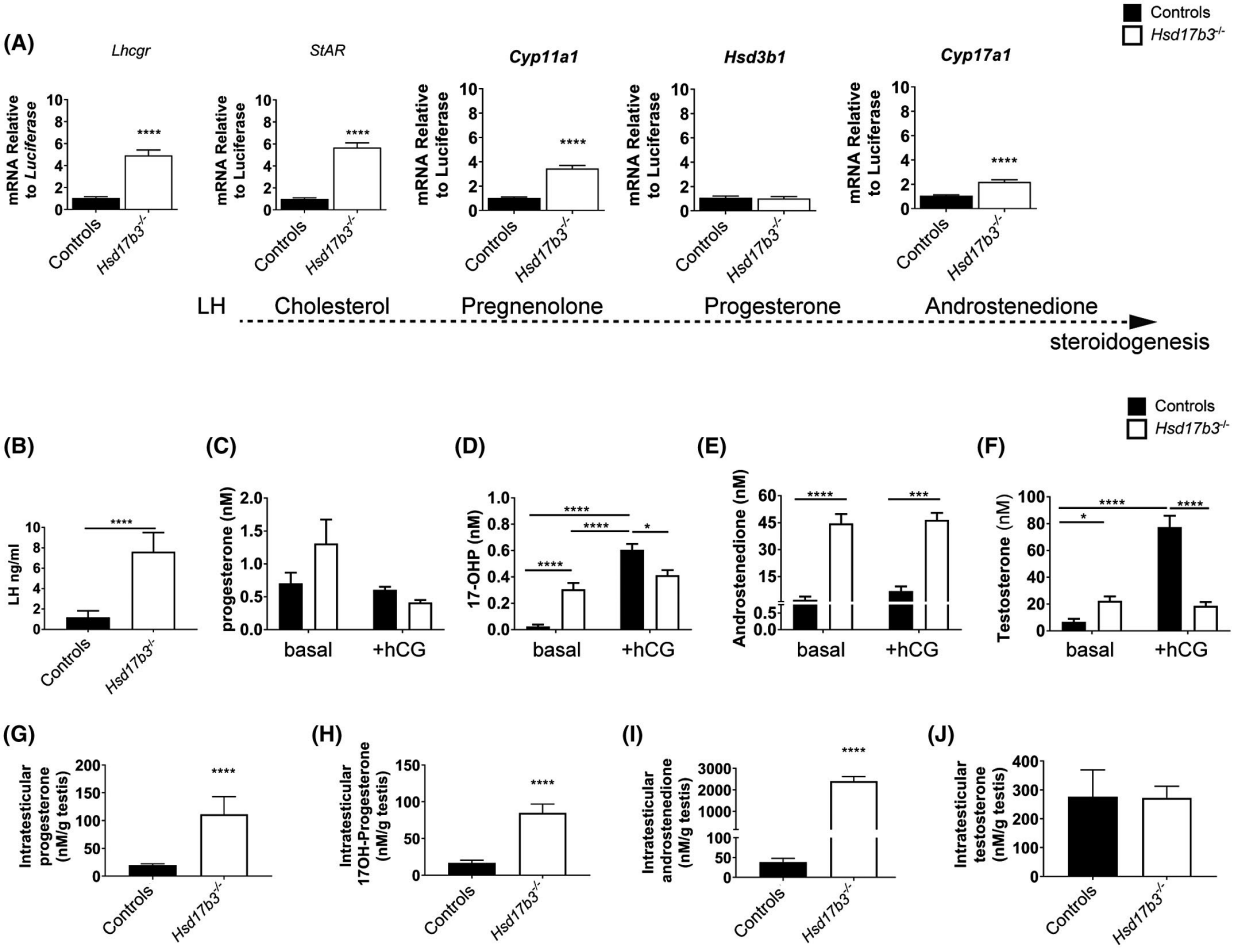
HSD17B3 loss of function impacts Leydig cell but not the intratesticular basal testosterone concentrations. Comparative testicular expression of Leydig cell (A) steroidogenic transcripts in adults (d80) (n = 14 controls and n = 8 *Hsd17b3* ^−/−^, T test ***P < .001, ****P < .0001). Circulating LH (B) was measured in controls vs *Hsd17b3* ^−/−^ and circulating (C) progesterone, (D) 17‐OH‐progesterone (17‐OHP) (E) androstenedione and (F) testosterone were measured in basal and hCG‐stimulated conditions in adult (d80) *Hsd17b3* ^−/−^ and controls littermates animals (n = 24 (basal) n = 11 (+hCG) and n = 12 (basal) n = 6 (+hCG) *Hsd17b3* ^−/−^, two‐way ANOVA, *P < .05, ***P < .001, ****P < .0001). Intratesticular hormones (G) progesterone, (H) 17‐OH‐progesterone (I) androstenedione and (J) testosterone in adult (d80) *Hsd17b3* ^−/−^ and control littermate animals (n = 10 controls and n = 11 *Hsd17b3* ^−/−^, T test and Mann Whitney, ****P < .0001) under basal conditions. Controls correspond to *Hsd17b3* ^+/+^ males pooled with *Hsd17b3* ^+/−^ **males

To understand how male mice lacking HSD17B3 apparently exhibit a near‐normal phenotype, we next analyzed the impact of ablation of HSD17B3 on Leydig cell function. Assessment of Leydig cell function in adult (d80) mice lacking HSD17B3 revealed a significant upregulation of *Lhcgr* transcripts in addition to transcripts encoding most of the steroidogenic enzymes that function in the canonical testosterone production pathway *StAR*, *Cyp11a1*, and *Cyp17a1* (Figure 4A). *Lhcgr*, *StAR*, *and* *Cyp11a1* are known to increase expression in response to increased LH stimulation,^21^ suggesting that there is significant hyper‐stimulation of the Leydig cell population in the *Hsd17b3* ^−/−^ animals. Measurement of the circulating hormone profile in these animals confirmed this, with circulating LH significantly increased in *Hsd17b3* ^−/−^ males compared to wild‐type males (Figure 4B).

Increased circulating LH is an indicator of possible compensated Leydig cell failure.^22^ To assess the impact of loss of HSD17B3 on Leydig cell function, we measured circulating concentrations of testicular steroids under basal or hyper‐stimulated conditions (+hCG) in adulthood (d80). Circulating progesterone concentrations did not differ between genotypes under basal or hyper‐stimulation conditions (Figure 4C), however, circulating 17‐OH‐progesterone (17‐OHP), androstenedione, and surprisingly, circulating testosterone, were all significantly higher in the *Hsd17b3* ^−/−^ males under basal conditions (Figure 4D‐F). 17‐OHP and testosterone failed to increase further upon hyperstimulation in *Hsd17b3* ^−/−^ males in contrast to controls, which showed increased levels. These data suggest that in the absence of HSD17B3, hCG‐mediated hyperstimulation is unable to increase testosterone production.

To investigate this further, we next determined the intratesticular concentrations of the same hormones. This revealed that, under basal conditions, progesterone, 17‐OHP, and androstenedione are all significantly increased in the testis of *Hsd17b3* ^−/−^ males (Figure 4G‐I), while testosterone is present in the same concentration as control littermates (Figure 4J).

Together these data suggest that HSD17B3 may act as a rate‐limiting step on testosterone production in response to hyperstimulation and, in its absence, the hypothalamus‐pituitary‐gonadal (hpg) axis responds in a manner consistent with compensated Leydig cell failure to maintain basal testosterone production via another pathway resulting in increased circulating LH levels in the presence of normal testosterone.

### 3.6 HSD17B3 acts as a rate‐limiting step in testicular testosterone production

To address this hypothesis, we sought to rescue the steroidogenic phenotype of the *Hsd17b3* ^−/−^ adult males by delivery of *Hsd17b3* cDNA to the testis via lentiviral‐mediated gene therapy. We first injected lentivirus into the interstitium resulting in the transduction of Leydig cells, but this also led to Leydig cell death within ten days (Figure S2), so we chose to instead deliver the lentivirus to adult Sertoli cells (rather than Leydig cells) in wild‐type and *Hsd17b3* ^−/−^ males using intra‐rete injection.^12^ In retrospect, this provided additional information because we were now able to determine (i) whether we could rescue the phenotype observed in *Hsd17b3* ^−/−^ males and (ii) whether the testis could be induced to function as a single testosterone‐producing unit in adulthood, analogous to the situation in the normal fetal testis.^3^, ^4^

**FIGURE 5:**
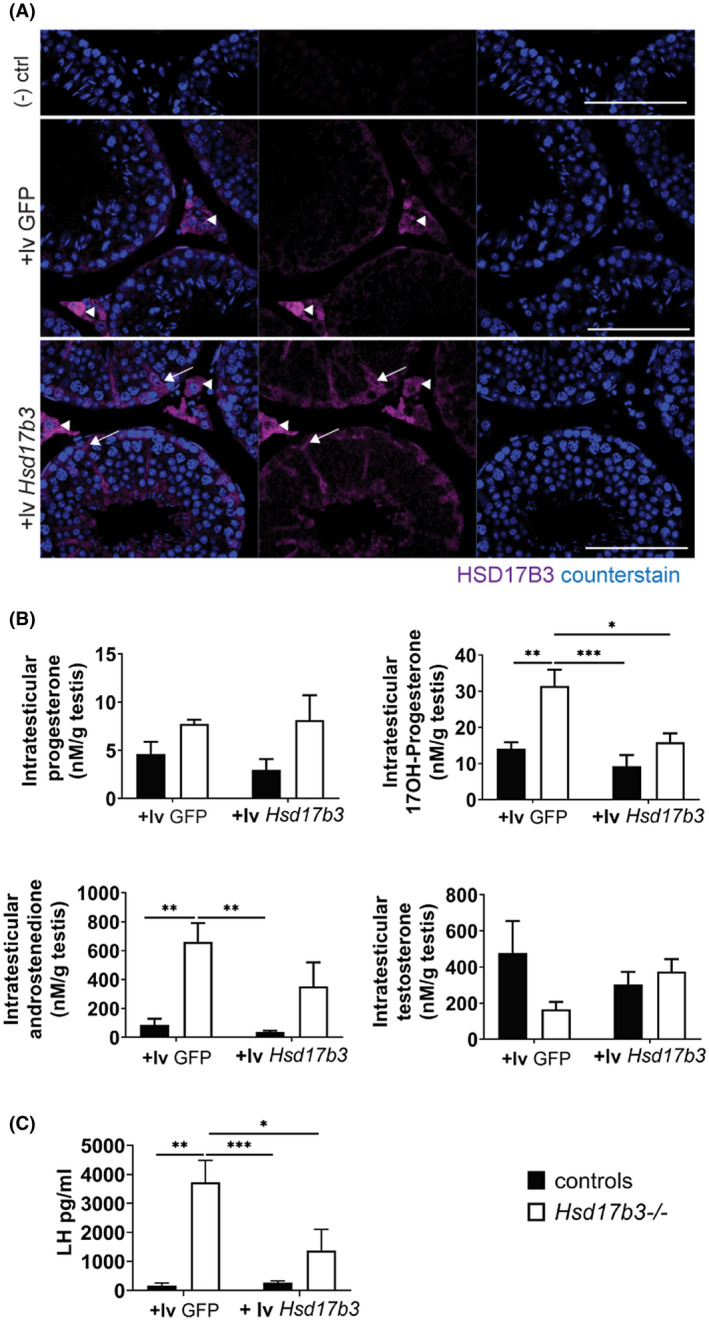
HSD17B3 acts as a rate‐limiting step in testicular testosterone production. A, Immunolocalization of HSD17B3 following the intra‐rete injection of control lentiviral particles (+lv GFP) and *Hsd17b3* lentiviral particles (+lv *Hsd17b3* (bar: 100 µm). Intratesticular hormones following intra‐rete injection of control lentiviral particles (+lv GFP) and *Hsd17b3* lentiviral particles (+lv *Hsd17b3)* that directs *Hsd17b3* expression into Sertoli cells (B) progesterone, 17‐OH‐progesterone, androstenedione, and testosterone in adult (d80) *Hsd17b3* ^−/−^ and controls littermates (n = 10 controls and n = 11 *Hsd17b3* ^−/−^ two‐way ANOVA, *P < .05, ** *P* < .01, ***P < .001) (C) Circulating LH levels (from tail vein) reduce following the reintroduction of *HSD17b3* (n = 4‐7 controls and n = 6 *Hsd17b3* ^−/−^, two‐way ANOVA, *P < .05, ** *P* < .01, ***P < .001). Controls correspond to *Hsd17b3* ^+/+^ males pooled with *Hsd17b3* ^+/−^ **males

To validate HSD17B3 transgene delivery and expression, cDNA constructs (*GFP* control, or *Hsd17b3*) enclosed in lentiviral particles were delivered via the rete testis at d120 and the testes recovered 7 weeks later (to permit recovery from surgery and completion of an entire cycle of spermatogenesis). Transgenic expression of HSD17B3 within Sertoli cells was confirmed by immunofluorescence (Figure 5A). We then repeated the study and processed the tissue to measure intratesticular steroid hormone concentrations, to determine the impact of restoring HSD17B3 function. Delivery of GFP lentivirus (lv GFP) did not modify the intratesticular levels of steroids away from those previously observed in untreated animals (as in Figure 4H‐K). However, in *Hsd17b3* ^−/−^ males treated with *Hsd17b3* cDNA (lv *Hsd17b3*), while testicular concentrations of progesterone remained unchanged, levels of 17‐OHP, and androstenedione were both reduced to levels observed in control littermates (Figure 5A‐B). Testosterone concentrations within the testis remained unchanged (two‐way ANOVA *P* value = .5648) (Figure 5A,B), however, circulating LH concentrations also fell to levels consistent with control littermates (two‐way ANOVA *P* value = .0010) (Figure 5B,C). Together these data show that restoration of HSD17B3 function is able to rescue the steroidogenic phenotype observed in HSD17B3^−/−^ animals and, also, that this can be achieved via gene delivery to the Sertoli cells, indicating that the testis can be manipulated to function as a single steroidogenic unit in adulthood. This also confirms that the role of HSD17B3 is to act as a rate‐limiting step in the testosterone production system in the testis and cannot be fully compensated for in this role by another enzyme. However, the mechanism underpinning basal testosterone production in the absence of HSD17B3 remains unknown.

### 3.7 Identification of AKR enzymes underpinning basal testosterone production

**FIGURE 6:**
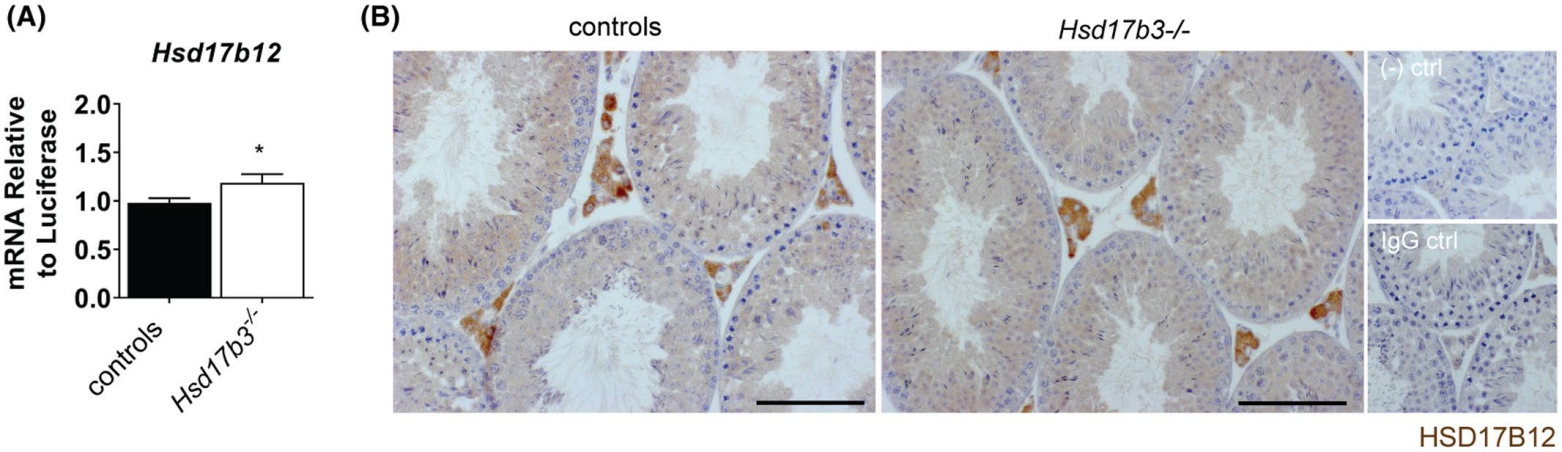
Expression of potential androgen converting AKR enzymes. Comparative testicular expression of (A) *Hsd17b12* transcript in adult (d80) (n = 14 controls and n = 8 *Hsd17b3* ^−/−^, *t*‐test *P < .05), B, HSD17B12 protein localizes to Leydig cells, with evidence of lower expression in germ cells in both *Hsd17b3* ^+/+^ (controls) and *Hsd17b3* ^−/−^ adult testis (bar: 100 µm). (−) ctrl stands for no primary control and IgG ctrl for IgG control

The production of testosterone in the absence of HSD17B3 suggests the presence of another aldo‐keto reductase enzyme (AKR) that performs this role in the testis. The other known HSD17Bs capable of converting androstenedione into testosterone are HSD17B1, HSD17B5,^4^, ^17^, ^23^, ^24^ and HSD17B12.^25^ When measured by qPCR (Figure S3, *Hsd17b1* and *Hsd17b*5 transcript levels were below the detection threshold in the testis of control and *Hsd17b3* ^−/−^ animals in adulthood. This is consistent with previous proteomic analysis of the adult mouse testis where both HSD17B1 and HSD17B5 are not detected.^26^ In contrast, *Hsd17b12* transcript levels were quantifiable and show a small but significant increase in expression following ablation of HSD17B3 (Figure 6A); HSD17B12 has previously been identified as a testis‐expressed protein in both mouse and human^26^ and immunohistochemistry localizes HSD17B12 to Leydig cells and more weakly in the germ cells in both control and Hsd17b3^−/−^ testes Figure 6B. As the only known AKR enzyme expressed in the mouse testis that is able to produce testosterone, other than HSD17B3, the significance of HSD17B12 for basal testosterone production in the testis requires further investigation.

## 4 DISCUSSION

To determine the role of the steroidogenic enzyme HSD17B3 in testosterone production and androgenization during male development and function, we characterized a mouse model lacking HSD17B3. These data reveal that developmental masculinization and fertility are normal in male mice lacking the enzyme widely accepted to be critical for the canonical testosterone production pathway. Ablation of *Hsd17b3* induces compensation of steroidogenic function within the testis to maintain normal testosterone levels. Reintroduction of *Hsd17b3* via gene‐delivery to Sertoli cells in adulthood rescues this compensation, showing that, as in development, different cell‐types in the testis can work together to produce testosterone. The data also confirm that HS17B3 acts as a rate‐limiting step in testosterone production but does not control basal testosterone production. Interrogation of other known aldo‐keto reductase (AKR) and short‐chain dehydrogenase/reductase enzymes (SDR) enzymes able to produce testosterone as a product suggest HSD17B12 as a candidate enzyme driving basal testosterone production in the testis in the absence of HSD17B3. The data show that testicular androgen production is a well‐protected process given its importance in masculinization and male fertility.

Testosterone is essential for masculinization of the male fetus,^27^, ^28^ and HSD17B3 is well established as the critical enzyme controlling the conversion of androstenedione to testosterone in the canonical testosterone production pathway in adulthood. Previous studies in mice^3^, ^4^ have shown that, during fetal life, androstenedione produced by fetal Leydig cells is converted to testosterone by Sertoli cells. Consistent with this, HSD17B3 is expressed in Sertoli cells during fetal life and likely acts in this role. However, our data reveal that HSD17B3 is, in fact, dispensable for basal testosterone production in fetal life, as *Hsd17b3* ^−/−^ mice are normally masculinized at birth. This is in contrast to humans lacking HSD17B3 function who are under‐virilized at birth.^11^ The explanation for this is unclear, but it does suggest the presence of another HSD17 enzyme able to support testosterone production in the mouse fetal testis. Recently, *Hsd17b1* expression was localized to Sertoli cells in mice in fetal life.^29^ Ablation of this enzyme leads to disruption of the seminiferous epithelium and abnormal spermatozoa in adulthood, linking it to a role in the establishment of normal male fertility.^29^ As *Hsd17b1* is able to convert androstenedione to testosterone this raises the possibility that HSD17B1 is responsible for basal androgen production in fetal life, though as it is not expressed in the adult testis, is unable to explain the normal testosterone concentrations observed in the *Hsd17b3* ^−/−^ adult males.

Phylogenetic analysis of the hydroxysteroid family in human highlights the clustering of HSD17B3 and HSD7B12 as well as HSD17B1 and HSD17B7.^30^ While the majority of the HSD17Bs share ~20% of amino acid sequence homology, HSD17B3 and HSD17B12 share 40% of similarity,^31^ supporting their overlapping activities. Further aligning to this, while ablation of *Hsd17b12* function is embryonic‐lethal in mice, heterozygous *Hsd17b12* males display reduced levels of androgens.^32^, ^33^ Our data also highlight *Hsd17b12* as a possible candidate for basal testosterone production in the fetal and/or adult testis, as it is expressed in the testis throughout life, and transcript levels are significantly increased in *Hsd17b3* ^−/−^ adult males. HSD7B12 in humans is present in Leydig cells and Sertoli cells^34^ and in “Human protein atlas” (www.proteinatlas.org)^35^ and has been identified in the proteome of whole adult mouse testis^26^, ^36^ and our own immunohistochemical analysis localizes HSD17B12 to Leydig cells and faintly in the germ cells in control and Hsd17b3^−/−^ testes. However, while *Hsd17b12* is able to convert androstenedione to testosterone in mice,^37^ its steroidogenic activity is largely restricted to estrone reduction to estradiol in humans, with low levels of androstenedione reduction.^25^, ^31^ Overall, such species differences could explain why we observe normal masculinization of *Hsd17b3* ^−/−^ mice; while reproductive tissues are impacted in humans lacking HSD7B3. Whether this is the case, or indeed whether this explains the apparent dispensability of *Hsd17b3* for basal testosterone production requires further investigation. A further explanation could be that, in humans, masculinization requires that both canonical and alternative (backdoor) androgen pathways are intact and the alternative pathway may also be dependent on a functional HSD17B3.^38^, ^39^ This alternative pathway does not appear to be of importance in fetal masculinization in the mouse.^40^

HSD17B3 is a key enzyme in the biosynthesis of testosterone and models where the androgen signaling pathway is impacted, (eg, *Tfm* mice which lack Leydig cell‐specific androgen receptor) have highlighted the importance of androgens for Leydig cell development.^41^, ^42^ While the number of Leydig cells in adult is normal, Leydig cell function is perturbed. Androgen production from puberty is under the regulation of the hypothalamic‐pituitary‐gonad axis and stimulation of LH induces the upregulation of its receptor and other crucial steroidogenic markers.^21^ This upregulation can be seen in *Hsd17b3* ^−/−^ males with significantly higher transcript levels of *Lhcgr*, *StAR*, *Cyp11a1*, and *Cyp17a1*. Increased LH and steroid levels (circulating and intratesticular) and the unresponsiveness to further hCG stimulation in the *Hsd17b3* ^−/−^ males is an indicator of possible compensated Leydig cell failure.^22^ The maturity stage of the Leydig cells has been shown to impact the responsiveness of the cell to stimulation. However, the increase of both circulating LH and testosterone would suggest the absence of proper feedback regulation in the hypothalamo‐pituitary‐gonad axis. Literature shows that the programming actions of testosterone on the brain can be mediated by androgenic or estrogen action due to aromatization of testosterone. A sex‐dependent regulation has also been suggested wherein androgens in males tend to inhibit the fetal neonatal gonadotrophin releasing hormone (GnRH) input compared to females and that daily treatment with GnRH agonist can impact the normal developmental changes in Leydig cell function.^43^, ^44^, ^45^ Kisspeptin signaling is a component of the neuroendocrine regulation of the reproductive system and a role for kisspeptin in mediating the negative feedback effects of gonadal steroids on GnRH secretion in both the male and female via the estrogen and androgen receptor has been described.^46^, ^47^ We speculate that in our *Hsd17b3* ^−/−^ model, the action of the high circulating androgens, regardless of aromatization, impact the kisspeptin neurons and GnRH secretion consequently altering the negative feedback, however, this requires further investigation.

While this data show that HSD17B3 is not strictly necessary for testosterone production or fertility in the mouse, the enzyme is necessary for optimal testosterone production above basal levels. In fetal life, the conversion of androstenedione, produced by fetal Leydig cells, to testosterone is undertaken by Sertoli cells.^3^, ^42^ In adulthood, in contrast, Leydig cells carry out the full canonical testosterone biosynthetic pathway while Sertoli cells act to maintain Leydig cell viability and function.^3^, ^4^, ^15^, ^48^ The reintroduction of *Hsd17b3* in Sertoli cells in *Hsd17b3* ^−/−^ males was able to partially rescue the endocrine defect in *Hsd17b3* ^−/−^ males, indicating that the somatic cells of the adult testis can act cooperatively to produce testosterone.

In conclusion, this study expands our knowledge of testosterone production in the mouse which has implication for our wider understanding of masculinization and male fertility. In addition, the ability to re‐express *Hsd17b3* in Sertoli cells and manipulate this cell type to produce testosterone suggests that Sertoli cells could be engineered to produce androgens and could form the basis of future therapy to combat the natural decline in androgens during aging.

## CONFLICT OF INTEREST

The authors declare no conflict of interest.

## AUTHORS CONTRIBUTIONS

Conceived and designed the study: D. Rebourcet, P.J. O’Shaughnessy, L.B. Smith Carried out experiments: D. Rebourcet; P.J. O’Shaughnessy; R. Mackay; A. Darbey; M. K. Curley; Analyzed the results: D. Rebourcet; R. Mackay; P.J. O’Shaughnessy; L.B. Smith Provided novel resources: S. Nef; R.T. Mitchell; A. Jørgensen; H. Frederiksen Wrote the paper: D. Rebourcet, P.J. O’Shaughnessy, L.B. Smith
