# Supplementary figures and images for: Ablation of the canonical testosterone production pathway via knockout of the steroidogenic enzyme HSD17B3, reveals a novel mechanism of testicular testosterone production
Source: FASEB J. 2020 Jun 18;34(8):10373–86. doi: 10.1096/fj.202000361R (PMC7496839; doi:10.1096/fj.202000361R)

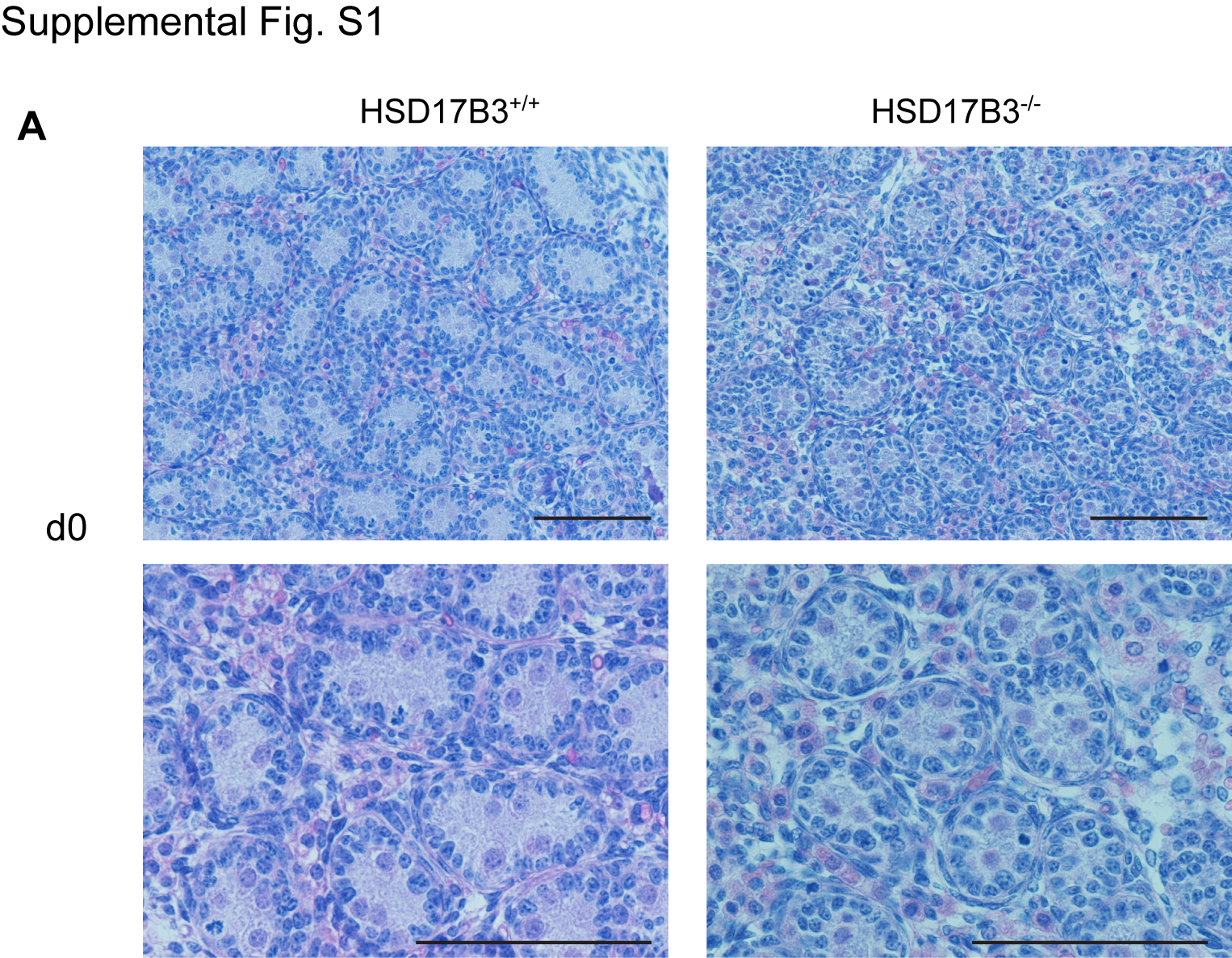

Supplement: Supplementary file 1 [file FSB2-34-10373-s001.tif]

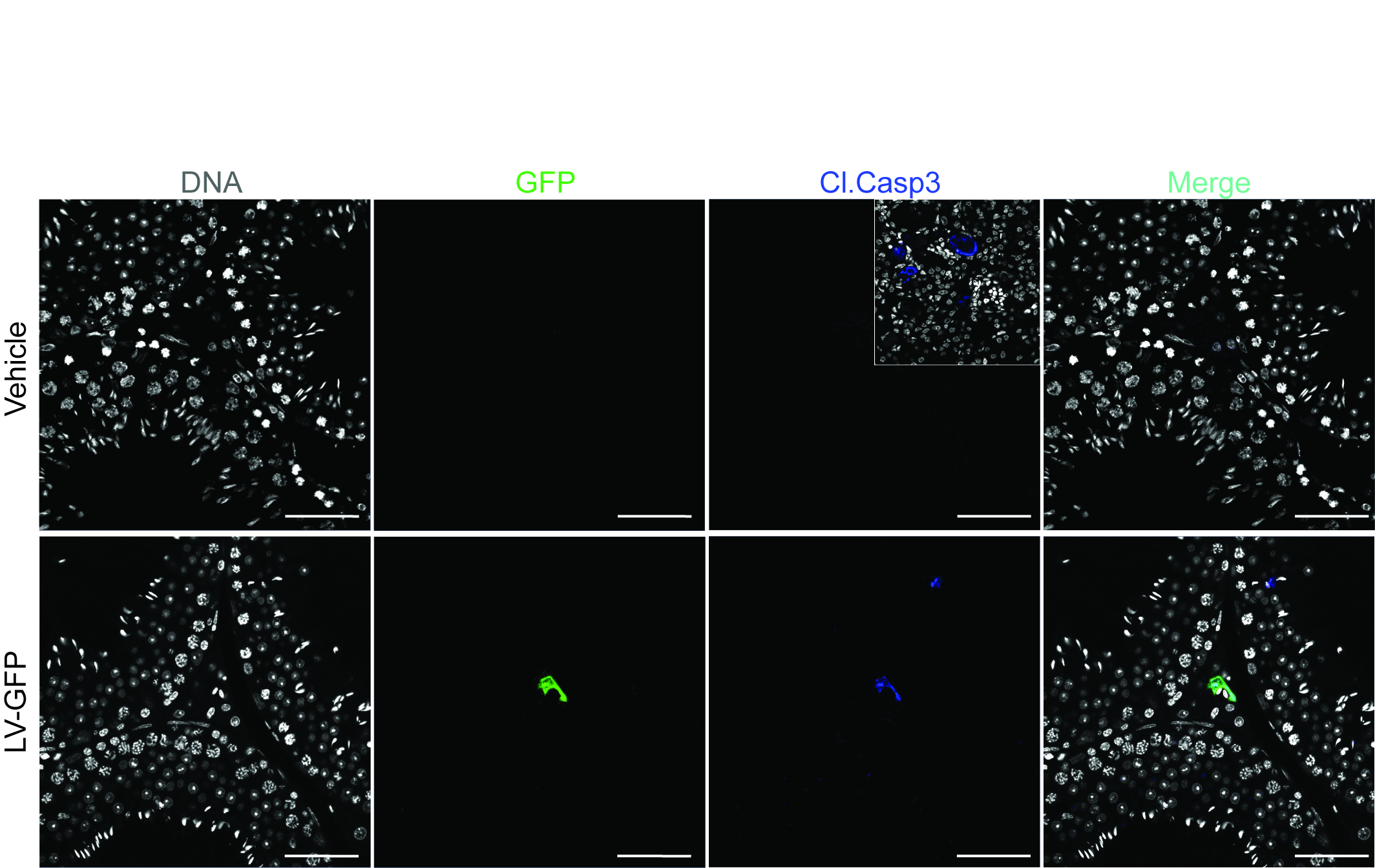

Supplement: Supplementary file 2 [file FSB2-34-10373-s002.tif]

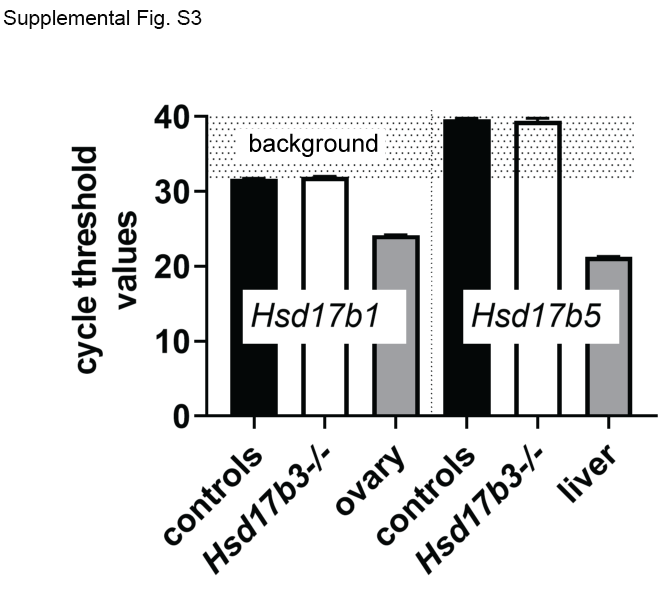

Supplement: Supplementary file 3 [file FSB2-34-10373-s003.tif]
