# Supplementary material for: Ablation of the canonical testosterone production pathway via knockout of the steroidogenic enzyme HSD17B3, reveals a novel mechanism of testicular testosterone production
Source: FASEB J. 2020 Jun 18;34(8):10373–86. doi: 10.1096/fj.202000361R (PMC7496839; doi:10.1096/fj.202000361R)
Supplement: Supplementary file 4 [file FSB2-34-10373-s004.docx]

**Supplemental Figure legends:**

**Supplemental Figure S1.** **HSD17B3 loss of function does not alter testis development in neonatal life**

Testicular histology was normal in *Hsd17b3-/-* compared to *Hsd17b3+/+* animals in neonatal life (d0) (bar: 100 µm).

**Supplemental Figure S2. Lentiviral-mediated gene therapy targeted to Leydig cells leads to Leydig cell death**

Adult testis injected with either vehicle or lentiviral vector (LV-GFP) carrying a GFP-expressing transgene immunostained for GFP (green) and Cl.Casp3 (blue) (Inset: Liver positive control, scale bars = 20μm).

**Supplemental Figure S3. *Hsd17b1* and *Hsd17b5* transcript levels are below detection threshold in the testis**

Level of detection (expressed in cycle threshold values) for amplification of *Hsd17b1* and *Hsd17b5* transcript level in testis and tissue positive controls.
